# Schistosomiasis: an epidemiological update on Brazil's southernmost low endemic area in Esteio

**DOI:** 10.1590/0037-8682-0411-2020

**Published:** 2020-10-05

**Authors:** Angélica da Paz Ramírez, Vivian Favero, Catieli Gobetti Lindholz, Carolina de Marco Veríssimo, Vanessa Fey Pascoal, Renata Russo Frasca Candido, Alessandra Loureiro Morassutti, Carlos Graeff-Teixeira

**Affiliations:** 1 Pontifícia Universidade Católica do Rio Grande do Sul, Escola de Ciências, Departamento de Ecologia e Biodiversidade, Porto Alegre, RS, Brasil.; 2 University of Western Australia, Department of Physics, Perth, Australia.; 3 Universidade Federal do Espírito Santo, Centro de Ciências da Saúde, Departamento de Patologia e Núcleo de Doenças Infecciosas, Vitória, ES, Brasil.

**Keywords:** Schistosomiasis, POC-CCA, Helmintex, MAMA, Esteio, Low endemicity

## Abstract

**INTRODUCTION:**

Brazil’s southernmost state, Rio Grande do Sul (RGS), was considered schistosomiasis-free until 1998 when a low endemic focus was identified in Esteio, a city located next to the capital of RGS. In the last two decades, the control interventions applied in the region have been apparently successful, and the absence of new cases indicated the possibility of interrupted schistosomiasis transmission. The objective of this study was to update the clinical and epidemiological data of schistosomiasis in Esteio.

**METHODS:**

We reviewed all 28 individuals diagnosed with the infection since 1997 and a survey was applied to a group of 29 school-aged children residing in Vila Pedreira, one of the most affected neighborhoods.

**RESULTS:**

No eggs were detected in fecal samples using the Helmintex method, and all samples were negative for serum antibodies on examination by the western blot technique using the *Schistosoma mansoni* microsomal antigen (MAMA- WB). In contrast, 23 individuals (79%) tested positive for the cathodic circulating antigen with the point-of-care immunochromatographic test (POC-CCA) on urine samples. Of the 28 formerly infected individuals, only eight were located, of which four tested positive, and four tested negative for serum antibodies using the MAMA-WB technique.

**CONCLUSIONS:**

Current adverse conditions for *S. mansoni* transmission in Esteio and the absence of a confirmed diagnosis suggests that there is (i) a lack of specificity of the POC-CCA test in low endemic settings, and (ii) a high probability that interruption of schistosomiasis has been achieved in Esteio.

## INTRODUCTION

The areas in Brazil endemic for schistosomiasis infection include six northeastern states, the southeastern state of Minas Gerais, and 12 additional states that were found to be the focus of transmission^1^ ^,^ ^2^. Located next to the border with Uruguay and Argentina, the state of Rio Grande do Sul (RGS) has been considered schistosomiasis-free; however, the autochthonous transmission of the parasite was confirmed in 1998 after the identification of infected snails at two sites next to the Sinos River in Esteio^3^. *Biomphalaria glabrata* snails were first documented in Esteio in 1997 after schistosomiasis was diagnosed in an adult male hospitalized with hepatitis. However, the occurrence of autochthonous infection was not confirmed^4^. The presence of active transmission was documented a year later after the identification of snails infected with *Schistosoma mansoni* ^3^, concurrent with the detection of a second individual diagnosed with schistosomiasis. In this study, we aimed to update the epidemiological data for the transmission of schistosomiasis and tested the hypothesis of transmission interruption in Esteio (RGS).

## METHODS

### Study areas and populations

The municipality of Esteio (29º51′ S, 51º10′ W) is located in the metropolitan region of Porto Alegre, the capital of Brazil’s southernmost state, RGS. The town of Esteio, which had an estimated population of 84,114 in the year 2016, is located alongside the Federal Road BR-116, in a north-south direction, to the eastern side of the highway^5^. The Sinos River runs in a north-south direction, where infected *B. glabrata* specimens were found in the swampy plains along the riverside in the following sites: (1) Banhado do Azeite (BA); (2) Valo da Três Portos (VTP); and (3) Casa dos Trilhos (CT). The site CT was an isolated shallow pond next to the Siderúrgica Riograndense railway, and snails were not detected at this location after the initial findings in 1999^6^. At the site Fazenda Kroeff (FK), there were indications for transmission, and snails were found, though the presence of *S.mansoni* in snails was never documented. VTP was the only area where people living next to the breeding sites of snails with active transmission last documented in 2011. Infected individuals were residents of the VTP, Novo Esteio (NE), and Vila Pedreira (VP) neighborhoods, where most of the individuals that were initially infected lived (**Table 1**).

### Collection of biological samples for the epidemiological survey at VP

Fecal, urine, and serum samples were collected from 29 asymptomatic school-aged children (aged 6 to 9 years) from the Centro Municipal de Educação Basica Trindade (Municipal Center for Basic Education Trindade), a community school in VP, between November to December 2015. The children examined had no history of travel to endemic areas or exposure to transmission sites near the Sinos River. The urine and blood samples were collected on a single day at school, and students were given instructions to collect their stools at home and bring it back to the school. Before the collection of biological material from the students, several activities were pursued in the classroom at the community school, including playing games, watching videos, observing live snails, viewing parasitic structures using the microscope, and having educational discussions about health and other issues. 

### Follow-up investigation of previously infected individuals

A search for previously infected individuals was performed by visiting their homes at the addresses registered when the patient was documented as being infected. Queries regarding the status of their general health and risky behavior were put to them. For individuals who had relocated, the neighbors/relatives were asked for the new addresses. Additionally, we reviewed the files at the Laboratory of Parasite Biology at The Pontifical Catholic University of RGS to summarize and update the information related to the tests performed for the infected individuals in Esteio.

### Enzyme-linked immunoelectrotransfer blot

Approximately 5 mL of blood was collected via venipuncture under aseptic conditions and transferred to 15 mL centrifuge tubes for the separation of serum on the same day. Serum samples were stored at -80°C for further use. Strips containing *S. mansoni* microsomal antigen (MAMA) and the protocol for western blot were obtained from the Centers for Disease Control and Prevention, USA^7^ ^,^ ^8^. Test sera were diluted 1:100 in PBS containing 0.3% Tween 20 (Sigma-Aldrich, USA) and 5% nonfat milk powder (Nestlé, Brazil) and incubated with the strips for 1 h at room temperature (RT) with agitation. After 4 washes with 0.3% Tween-PBS, the strips were incubated with anti-human IgG coupled to peroxidase for 1 h at RT, given 4 washes with 0.3% Tween-PBS, and were subsequently developed using 3,3’-Diaminobenzidine (Sigma-Aldrich, USA). Positive and negative control sera were included for each batch of strips.

### Detection of antigen in urine

Urine samples collected in VP were refrigerated and tested in the laboratory on the same day. Point-of-care circulating cathodic antigen test (POC-CCA; Rapid Medical Diagnostics, Pretoria, South Africa; lot number: 50182, expiry date: 2017/10) was performed as per the manufacturer’s instructions. Briefly, one drop of urine was placed in the cassette, followed by a drop of the supplied buffer. After 20 min, three observers independently checked for the presence of the control and test lines. A “trace” was defined as a faint line without clearly defined margins. 

###  The Helmintex method (HTX) for the detection of *S. mansoni* eggs 

The HTX assay was performed according to the technique described by Favero and collaborators^9^. The stool samples (30 g) were fixed in a solution of 5% Tween-20/70% ethanol. After fixation and homogenization, the fecal suspension was sieved through a 500 μm metal mesh, transferred to a conical flask, and washed until a clear supernatant was obtained. The resulting sediment was further sieved through metal meshes with openings of 150 μm and 45 μm sequentially, and the latter sieving process was performed to retain the *S. mansoni* eggs. The fraction retained by the last sieve (45 μm) was suspended in a 30% (v/v) ethyl acetate aqueous solution, homogenized, and centrifuged for 10 min at 200 ×*g*. After discarding the supernatant and the ring of debris, the pellet was transferred to a microtube containing 19 µL of paramagnetic iron oxide particles (Bangs Labs, USA). The microtubes were homogenized by orbital rotation for 30 min and placed on a magnetic rack (Bangs Labs, USA). After 3 min, the unbound material was discarded before removing the tubes from the rack. The sediment was re-suspended in 100 μL of 0.9% aqueous NaCl solution and stored at -4°C for further analysis. For microscopy analysis, the sediments were stained with 3% (w/v) ninhydrin (Sigma-Aldrich, USA) in 70% ethanol and evenly spread over 24 μm pore filter paper (UNIFIL, Brazil) of area 5 × 2.5 cm, provided an ID and kept for examination by optical microscopy (100× magnification).

### Ethical considerations

The research was conducted according to Brazilian regulations and conformed to the principles outlined in the Declaration of Helsinki of 1964 as revised in 2000. The study protocol was approved by the Research Ethics Committee of PUCRS (CAAE 18944614.3.0000.5336).

## RESULTS

### Evaluation of formerly infected individuals

Between 1997 and 2000, 11 infected individuals from Esteio were diagnosed as having schistosomiasis^6^, and an additional 17 individuals were found to be infected between 2001 and 2011 (Table 1). An updated summary of the clinical and epidemiological data for the 28 infected individuals is presented in Table 2. The patients were living in three neighborhoods: VP, NE, and VTP, at the time of diagnosis. Table 3 summarizes the information on the updated (2015) dwellings of the patients; information for 10 individuals was not available, 5 were living in the same place, and 13 individuals had moved from their original place of residence (1 had moved within, and 12 had relocated out of Esteio). Serum samples for follow-up experiments were collected from 8 formerly infected individuals, of which four each tested positive and negative using the MAMA-WB assay. No other method was used for the examination of these individuals.

**TABLE 1:** Distribution of the dwellings of 28 infected individuals in three neighborhoods: Vila Pedreira (VP), Novo Esteio (NE), and Valo da Três Portos (VTP), between 1997 to 2011, in Esteio, Rio Grande do Sul, Brazil.

Local/Year	97	98	99	00	01	02	03	04	05	06	07	08	09	10	11
VP	●●			●●		●	●●								
NE		●●●		●	●	●	●●					●		●	
VTP		●		●●							●●●		●●		●●●

**TABLE 2:** Summary of the original and updated clinical and epidemiological data for the 28 infected individuals from Esteio, Rio Grande do Sul, Brazil, who were infected between 1997 to 2011.

Id*	Date	Initial clinico-epidemiological situation	Neighborhoods	Follow-up in 2015	
				WB-MAMA	Clinical - epidemiological situation
**1**	January 1997	hepatitis, admitted to the local Hospital	Vila Pedreira	Positive	Asymptomatic
2	October 1997	positive in parasitological survey	Vila Pedreira	Negative	Asymptomatic
3	October 1998	urticaria, outpatient clinic	Novo Esteio	-	Moved, outbound Esteio
4	October 1998	sharing risk behavior with patient 3 (BA)	Novo Esteio	-	Not found
5	October 1998	sharing risk behavior with patient 3 (BA)	Novo Esteio	-	Moved, outbound Esteio
6	October 1998	positive in parasitological survey	VTP**	-	Not found
7	May 2000	positive in parasitological survey	Vila Pedreira	Positive	Asymptomatic
8	May 2000	positive in parasitological survey	Vila Pedreira	-	Not found
9	July 2000	Abdominal pain	VTP	-	Not found
**10**	October 2000	positive in parasitological survey	VTP	Positive	Asymptomatic
**11**	October 2000	positive in parasitological survey	Novo Esteio	-	Not found
**12**	October 2002	positive in serological survey, confirmed parasitologically	Novo Esteio	-	Moved to Montenegro, RS. Outbound Esteio
**13**	October 2002	positive in serological survey, confirmed parasitologically	Vila Pedreira	-	Death, not related to schistosomiasis
**14**	January 2003	positive in serological survey, confirmed parasitologically	Vila Pedreira		Moved to Canoas, RS. Outbound Esteio
**15**	June 2003	positive in serological survey, confirmed parasitologically	Novo Esteio	-	Moved to Imbé, RS. Outbound Esteio
**16**	Jullho 2003	positive in serological survey, confirmed parasitologically	Novo Esteio	Negative	Asymptomatic
**17**	Jullho 2003	positive in serological survey, confirmed parasitologically	Vila Pedreira	Positive	Asymptomatic
**18**	June 2001	positive in serological survey, confirmed parasitologically	Novo Esteio	-	Not found
**19**	July 2007	sharing risk behavior with patient 10 (VTP)	VTP	-	Not found
**20**	July 2007	sharing risk behavior with patient 10 (VTP)	VTP	-	Not found
**21**	November 2007	positive in parasitological survey	VTP	-	Not found
**22**	October 2008	positive in parasitological survey	Novo Esteio	-	Not found
**23**	June 2009	positive in parasitological survey	VTP	-	Not found
**24**	June 2009	positive in parasitological survey	VTP	-	Not found
**25**	July 2010	sharing risk behavior with patient 18 (BA)	Novo Esteio		Not found
**26**	August 2010	sharing risk behavior with patient 10 (VTP)	Canoas	Negative	Asymptomatic
**27**	November 2010	sharing risk behavior with patient 10 (VTP)	Canoas	Negative	Asymptomatic
**28**	November 2010	sharing risk behavior with patient 10 (VTP)	Unknown	-	Not found

*Id: identification code; ** **VTP:** Valo da Três Portos neighborhood.

**TABLE 3:** Updated (2015) information for the dwellings of the 28 individuals from Esteio, RS, Brazil who were infected with schistosomiasis between 1997 to 2011.

Information on residence	Number	Number
Known		18
Not moved	5	
Moved, outbounds Esteio	12	
Moved, inbounds Esteio	1	
Unknown		10
**Totals**	**18**	**28**

### School-aged children

Samples for urine, blood, and/or feces were collected from 29 children aged between 6 and 9 years (average 7.13 ± 0.97 yrs.), who had a gender distribution of 55% male and 45% female children. HTX and MAMA-WB assays were not performed on every sample because a few children showed resistance to submitting blood or feces, or both. Seven children provided only fecal and urine samples, while 16 children provided blood and urine samples alone (Table 4). No fecal or serum samples were found positive after examination by HTX and/or MAMA-WB assays; however, the POC-CCA urine test results were positive or a trace line was seen in 23 out of 29 samples (79.3%). Trace results for the POC-CCA test were observed in 8 of the 23 positive samples (considered as positive CCA detection, according to the manufacturer’s instructions), and 6 of the total 29 samples were CCA-negative (Table 4).

**TABLE 4:** A comparison of the results from three diagnostic methods: point-of-care immunochromatographic detection of circulating cathodic antigen (POC-CCA), Western-blot with microsomal *Schistosoma mansoni* antigen (WB-MAMA) and Helmintex for egg detection in feces. The data are from 29 school-aged children from Vila Pedreira, Esteio, Brazil, 2015.

Sample	POC-CCA	WB-MAMA	Helmintex
1	Positive	Negative	Negative
4	Positive	Negative	Negative
6	Positive	Negative	Negative
19	Positive	Negative	Negative
20	Positive	Negative	Negative
17	Positive	Negative	-
26	Positive	Negative	-
28	Positive	Negative	-
5	Positive	-	Negative
9	Positive	-	Negative
12	Positive	-	Negative
13	Positive	-	Negative
21	Positive	-	Negative
22	Positive	-	Negative
27	Positive	-	Negative
7	Trace	Negative	-
8	Trace	Negative	-
11	Trace	Negative	-
14	Trace	Negative	-
16	Trace	Negative	-
23	Trace	Negative	-
24	Trace	Negative	-
29	Trace	Negative	Negative
2	Negative	Negative	-
3	Negative	Negative	-
10	Negative	Negative	-
15	Negative	Negative	-
18	Negative	Negative	-
25	Negative	Negative	-

## DISCUSSION

The focus of schistosomiasis transmission in Esteio has several peculiar characteristics. First, *B. glabrata* snails have never been detected in Brazil’s southernmost state, RGS^4^ ^,^ ^10^, and these have probably been recently introduced in Esteio by fishermen to be used as baits, as suggested by several residents. Endemic areas in the northern state of Paraná, 1,100 km away from Esteio, have been the southern limit for the occurrence of *B. glabrata* in Brazil^1^. Second, swampy sites with infected snails were found to be sharply delimited next to the Sinos River and were located at some distance from the urbanized neighborhoods, especially in NE, west of BR-116 road, which had ~5,000 inhabitants. In VTP, a small number of families lived next to an old man-made channel (“*valo*”) to pump water from the river to a nearby cellulose industry named “Três Portos”. Between 1997 to 2011, the VTP area alone had overlapping houses and transmission sites before environmental changes and population reduction occurred, as described below. The Vila Pedreira neighborhood located immediately to the east of the BR-116 road had ~1,500 inhabitants and also had infected individuals. In Esteio, except for the inhabitants of VTP, exposure to *S. mansoni* infection may have been hindered by the long distances which require travel on foot, which may partially explain the ultra-low endemicity observed for more than two decades. Positivity rates were observed to be below 0.13% in surveys from 1997 to 2000^6^. As indicated in Table 1, most of the infected individuals initially resided in VP, and in 2011, most of the infected individuals were from VTP. 

The main transmission site was found to be Banhado do Azeite (BA), an approximately 48,000 m^2^ grassy shallow swamp on the left margin of the Sinos River. It was a popular fishing location used mainly by adults and young males, who commonly used snails as bait. People from the NE and VP communities, and others, reported that the areas next to the Sinos River were considered unsafe because of violence and activities related to drugs, as the river is used as an escape route to hide. Environmental modification has also contributed to the reduced transmission in BA and VTP. Since 2003, successful drainage treatment has prevented the land in BA from holding water, and consequently, it remains mostly dry. The occurrence of transmission was documented in VTP until 2011, but the construction of BR-448 road (between 2011 to 2013) affected the area, reducing its water content. All residents relocated from VTP after several conflicts, and a new owner started earthworks to build a warehouse. Thus, a combination of the geographic characteristics, environmental changes, and cultural-educational factors contributed to the decline in the transmission of schistosomiasis in Esteio. 

Further, based on the information provided by the children, their parents, and relatives, a striking change in behavior was observed concerning exposure to transmission sites next to the Sinos River, although these anecdotes were not part of the systematized data gathering procedure. The School Trindade in VP has made continuous efforts to build awareness in children and the community about behavioral risks and prejudices related to schistosomiasis. This would partly explain the negative results obtained from the serological and HTX assays in the 6 to 9 yr age group. Positivity rates in school-aged children (SAC) are usually a good indicator of the prevalence of schistosomiasis in the community at large. Further, testing SAC can be used to infer active transmission, and help avoid false-positive results from chronically infected, albeit cured adult individuals^11^. Additionally, this group is a priority target for control interventions^12^.

The MAMA assay was employed for the detection of antibodies and it has an estimated sensitivity and specificity of 99%^13^ ^,^ ^14^. Microsomal antigens, including those for other *Schistosoma* species such as *S. japonicum* (JAMA) and S. *haematobium* (HAMA), are useful for monitoring the prevalence of the disease, especially in areas of low endemicity^15^ ^,^ ^16^. Further, serum antibodies are long-lasting and can give positive results in patients who no longer carry the infection^17^.

Helmintex is a new egg-detection reference method for the identification of true-positive schistosomiasis-infected individuals. It has a 100% sensitivity for egg burdens higher than 1.3 eggs per gram of feces^18^, and is based on the isolation of eggs from a large volume of feces through their interaction with paramagnetic particles that can be recovered using a magnetic field^9^ ^,^ ^19^.

Antigen detection in urine is a promising alternative as a screening tool for the Kato-Katz method in areas with high endemicity^20^ ^,^ ^21^. In contrast, the performance of POC-CCA may be unreliable in low endemicity areas^22^ ^-^ ^25^. In our study, we obtained positive results from the POC-CCA test in 79.3% samples, with no corresponding positive results from the MAMA-WB and HTX assays, suggest that the test has potential specificity limitations. The confirmation of false-positives in the POC-CCA test requires the study of larger groups and the daily repetition of the HTX assay. However, this was not possible in 2015 due to operational difficulties resulting from the socially problematic settings in these areas. The low specificity of 35.5% was estimated for the POC-CCA test in a large population in the endemic area of southern Sergipe in Brazil^24^. Specificities for the POC-CCA test were evaluated to be higher in non-endemic areas in Tanzania (86.7%), Ethiopia (99%), and Ecuador (100%)^26^ ^-^ ^28^. Coelho and collaborators have demonstrated the inaccuracy of the “trace” results obtained using the POC-CCA test (considered as positive per the manufacturer’s instructions): after lyophilization, samples that previously showed ambiguous results in the POC-CCA test were found to be either positives or negatives^29^. The cost of such an improvement in the interpretation of results is the loss of the intended “point-of-care” character of the test^23^. The discrepancies in the accuracy of different batches of POC-CCA kits require resolution before additional extensive evaluations lead to the detection of antigen consistently and reproducibly in urine, especially in areas with low endemicity^22^ ^,^ ^23^ ^,^ ^30^.

In this study, the mobility of the population living next to the Sinos River schistosomiasis focus (**Table 3**) prevented the close follow-up of infected individuals by public health agents or research groups. For many individuals from the area, changing their address, and concealing information is part of a strategy to cope with socio-economic problems. However, although mobility can increase the probability of schistosomiasis spread, it may involve distancing from the transmission sites. Moreover, the active role played by community health agents (*agentes comunitários de saúde*) has considerably reduced and prevented the spread of schistosomiasis through the evaluation of risk behavior, providing immediate treatment, and close follow-ups.

In this study, there were difficulties in obtaining biological material, especially feces. This was a major drawback in many surveys undertaken in Esteio for two decades. In contrast, obtaining urine samples is much easier; and these can be obtained immediately after the first contact with households or at gatherings like the one established at the Trindade School for this study. However, the poor performance and reproducibility of the POC-CCA detection method is an important concern for its implementation as a routine screening procedure, especially in areas such as Esteio that have low endemicity^23^ ^,^ ^25^ ^,^ ^30^.

To conclude, there are indications that schistosomiasis transmission has been interrupted in Esteio, though the confirmation of this observation requires long-term monitoring. However, it is challenging to avoid a tendency to neglect epidemiological vigilance for a health problem that does not translate into a detectable disease. The current situation described in Esteio indicates a case of successful schistosomiasis control, that contributes to the national and global efforts of the Brazilian Ministry of Health, the Pan-American Health Organization, and the World Health Organization in containing the spread of schistosomiasis^12^ ^,^ ^31^.
